# Advances in functional magnetic resonance imaging-based brain function mapping: a deep learning perspective

**DOI:** 10.1093/psyrad/kkaf007

**Published:** 2025-04-29

**Authors:** Lin Zhao

**Affiliations:** School of Computing, University of Georgia, Athens 30602 GA, USA

**Keywords:** fMRI, brain function mapping, deep learning

## Abstract

Functional magnetic resonance imaging (fMRI) provides a powerful tool for studying brain function by capturing neural activity in a non-invasive manner. Mapping brain function from fMRI data enables researchers to investigate the spatial and temporal dynamics of neural processes, providing insights into how the brain responds to various tasks and stimuli. In this review, we explore the evolution of deep learning-based methods for brain function mapping using fMRI. We begin by discussing various network architectures such as convolutional neural networks, recurrent neural networks, and transformers. We further examine supervised, unsupervised, and self-supervised learning paradigms for fMRI-based brain function mapping, highlighting the strengths and limitations of each approach. Additionally, we discuss emerging trends such as fMRI embedding, brain foundation models, and brain-inspired artificial intelligence, emphasizing their potential to revolutionize brain function mapping. Finally, we delve into the real-world applications and prospective impact of these advancements, particularly in the diagnosis of neural disorders, neuroscientific research, and brain–computer interfaces for decoding brain activity. This review aims to provide a comprehensive overview of current techniques and future directions in the field of deep learning and fMRI-based brain function mapping.

## Introduction

Exploring and understanding the mechanisms of human brain function and its organization have been intense interests in the field of neuroscience for centuries (Brodmann, 1909; Belliveau *et al*., 1991; Raichle *et al*., 2001; Biswal *et al*., 2010). Since the early 1990s, functional magnetic resonance imaging (fMRI) based on blood-oxygen-level-dependent (BOLD) contrast has emerged as a dominant tool for imaging-based brain function research (O'Craven *et al*., 1999; Logothetis *et al*., 2001; Bassett and Bullmore, 2006; Logothetis, 2008). By imaging changes in blood flow and oxygenation levels (hemodynamic response), BOLD fMRI establishes an indirect mapping of neural activity and offers an *in vivo*, non-invasive tool to study the complex and dynamic processes of the brain (Heeger and Ress, 2002; Logothetis *et al*., 2001). Uncovering meaningful functional patterns and accurately mapping brain activity from the rich information contained in BOLD fMRI signals has since become a major focus for researchers in this field.

After decades of research, it has been revealed that human brain function is organized through the interaction of multiple concurrent neural networks, commonly referred to as functional brain networks (FBNs) (Von Der Malsburg, 1994; Cordes *et al*., 2000; Van Den Heuvel and Pol, 2010; Power *et al*., 2011). These FBNs distributed across specific neuroanatomical regions have become an established representation of brain function organization. Various methods have been developed to identify and reconstruct FBNs from noisy fMRI data, including the general linear model (GLM) for task-based fMRI (tfMRI) (Friston *et al*., 1994; Worsley, 1997), independent component analysis (ICA) for resting-state fMRI (rsfMRI) (Calhoun and Adali, 2006; Calhoun *et al*., 2009), and sparse dictionary learning (SDL) for both tfMRI and rsfMRI (Lv *et al*., 2014, 2015). The number of FBNs reconstructed from fMRI data is limited by the linear components in GLM and brain sources in ICA. In contrast, SDL, through an over-complete dictionary, can decompose fMRI data into hundreds or thousands of concurrent FBNs, offering a more detailed mapping of brain function.

Compared with traditional methods, deep learning has proven to be a powerful representation technique across various domains (LeCun *et al*., 2015; Goodfellow *et al*., 2016; He *et al*., 2016; Vaswani *et al*., 2017). Deep learning models such as convolutional neural networks (CNNs) (LeCun *et al*., 1995) and recurrent neural networks (RNNs) (Hochreiter and Schmidhuber, 1997) have been widely used to capture the complex spatiotemporal dynamics in fMRI data, offering robust representations of brain function. For example, CNNs have effectively identified individual FBNs from fMRI data using supervised learning (Zhao *et al*., 2017b, 2018c), while convolutional autoencoders excel at learning temporal patterns and spatial maps of brain activity in an unsupervised manner (Huang *et al*., 2017; Zhao *et al*., 2021). Recurrent units, such as long short-term memory (LSTM) networks (Hochreiter and Schmidhuber, 1997), have been extensively applied to model the temporal correlations in fMRI time series (Li *et al*., 2019, 2020). In addition to these established models, neural architecture search (NAS) methods (Zoph and Le, 2016; Liu *et al*., 2018) have been employed to autonomously discover neural network architectures optimized for aligning with the functional architecture of the human brain, offering new pathways for understanding and modeling brain function (Zhang *et al*., 2019b; Li *et al*., 2021a, b).

Recently, transformers and the self-attention mechanism have revolutionized artificial intelligence (AI) (Vaswani *et al*., 2017), driving the development of large-scale language/visual foundation models such as GPTs (Radford *et al*., 2018; Achiam *et al*., 2023). These advancements have also extended to brain functional mapping, where the self-attention mechanism, with its ability to capture long-range relationships across spatial and temporal dimensions, proves particularly effective for modeling complex neural dynamics and interactions (Zhao *et al*., 2022, 2023c). The brain foundation models are emerging as powerful tools by applying the same principles to large-scale neuroimaging datasets, enabling generalizable representations of brain activity that can be fine-tuned for various cognitive tasks (Thomas *et al*., 2022; Ortega Caro *et al*., 2024; Yang *et al*., 2024). The representation of brain function from fMRI is not confined to FBNs. Traditional FBN representations, which can be regarded as one-hot embeddings, may lack the nuance required to capture the full complexity of brain function. By contrast, embedding brain activity as dense vectors offers a more refined, richer and precise modeling representation of neural dynamics (Zhao *et al*., 2023a, c). This approach further allows for aligning the semantic representations of the human brain with the semantic representations of AI models, providing deeper insights about the mechanisms underlying intelligence (Zhao *et al*., 2023a; Liu *et al*., 2023b; Zhou *et al*., 2023).

In this review, we focus on the representation and mapping methods of brain function using deep learning techniques and fMRI data, aiming to identify key trends, challenges, and emerging opportunities in this rapidly evolving field. Rather than merely summarizing existing methods, we seek to highlight gaps in the current literature and propose new research directions to advance the field. This review is organized as follows: Section 2 elaborates on the various network architectures employed in the representation of brain function, including CNNs, RNNs, transformers, and methods involving NAS. Section 3 details the training schemes utilized in these studies, differentiating between supervised, unsupervised, and self-supervised training approaches. Section 4 summarizes current research trends, challenges, and future directions in the context of large foundation models. We conclude the review by discussing the current and potential impacts of these advanced techniques on neuroscience and clinical applications.

## Network architectures for brain function representation

In this section, we will delve into the various deep learning network architectures that have been employed to represent and map brain function using fMRI data. Each architecture brings unique strengths, enabling the capture of different aspects of brain activity. We will review the fundamental concepts, architectural designs, and specific applications of several prominent network architectures, such as CNNs, RNNs, transformers, deep belief networks (DBNs), graph neural networks (GNNs), and NAS techniques.

### Convolutional neural networks

CNNs have revolutionized the field of computer vision by enabling automatic feature extraction and hierarchical pattern recognition, significantly improving performance in tasks such as image classification, object detection, and segmentation (LeCun *et al*., 1995; He *et al*., 2016). With inductive biases, CNNs can effectively capture the spatial structure and hierarchies in 1D time series, 3D/4D image data. fMRI data are inherently 4D, consisting of three spatial dimensions and one temporal dimension. Each fMRI scan generates a series of volumes over time, capturing changes in blood flow and oxygenation levels. The time series extracted from each voxel is 1D, while considering the spatial volume at each time point provides a 3D representation. Treating the entire fMRI scan as a whole maintains its original 4D form. This multi-dimensional nature of fMRI data allows for the application of 1D, 3D, and 4D CNNs.

#### 1D convolutional neural networks

1D CNNs are specifically designed to handle sequential data. In the context of fMRI, each voxel's time series data can be treated as a 1D sequence, where the temporal dimension is the primary focus. By applying convolutional filters along the temporal axis, 1D CNNs are able to learn temporal dependencies and patterns within the time series data.

When applied to fMRI data, the architecture of 1D CNNs typically includes multiple convolutional layers, each followed by activation functions and pooling layers (Huang *et al*., 2017; Zhang *et al*., 2018b; Zhao *et al*., 2021, 2024). This hierarchical structure enables the network to capture both low-level, high-frequency temporal features and high-level, low-frequency temporal features. However, high-level features are often multi-dimensional and abstract, making them difficult to interpret. To address this, specific designs such as Feature Interpreter (Zhao *et al*., 2021, 2024) have been developed to elucidate the hierarchical features of fMRI time series.

**Figure 1:**
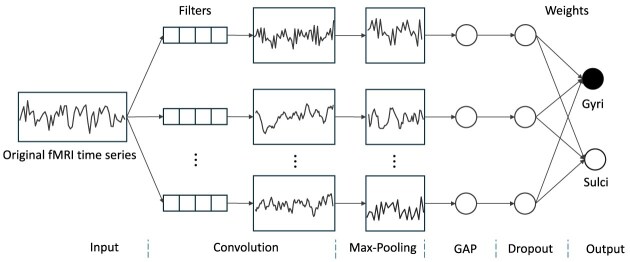
The architecture of the 1D CNN model proposed in Liu *et al*. (2019). This model is composed of a sequence of layers, including a convolutional layer, a max-pooling layer, a global average pooling (GAP) layer, a dropout layer, and a softmax layer. Each row in the convolutional layer corresponds to a different convolutional channel, and there are correspondences between the filters and weights in the model.

Alternatively, some approaches employ only a single convolutional layer, allowing the extracted features to directly correlate with the temporal characteristics of the fMRI time series (Liu *et al*., 2019; Wang *et al*., 2023). For instance, Liu *et al*. (2019) utilized a one-layer 1D CNN model to classify the fMRI time series from gyri and sulci of ceberal cortex (Fig. 1). By examining the weights of the fully connected layer that generates the predictions, they identified filters corresponding to gyri and sulci and analyzed them to explore the unique characteristics of these signals (Liu *et al*., 2019).

Moreover, 1D CNNs are less prone to overfitting due to the extensive amount of time series data in each 4D volume. In contrast, 3D and 4D CNNs often face challenges that the number of parameters is greatly larger than the number of data samples.

#### 3D convolutional neural networks

**Figure 2:**
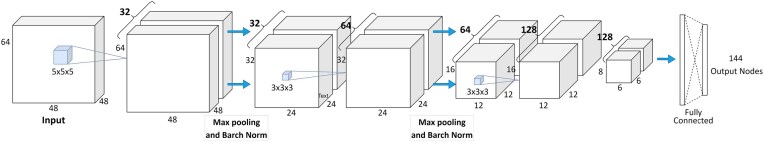
The architecture of the 3D CNN model proposed in Zhao *et al*. (2018a).

3D CNNs extend the convolutional operations to three dimensions, making them particularly suitable for analyzing volumetric data such as 3D volumes in an fMRI scan. 3D CNNs have been widely utilized in fMRI data analysis and FBN recognition. For instance, methods such as ICA and SDL can effectively reconstruct dozens or hundreds of FBNs from whole-brain fMRI signals, despite the lack of direct correspondences for these networks across different subjects. 3D CNNs can classify and recognize these derived FBNs (Ren *et al*., 2017; Zhao *et al*., 2018a) (Fig. 2), and have also been applied to differentiate autism spectrum disorder (ASD) by analyzing the 3D overlap patterns of FBNs (Zhao *et al*., 2018b). These networks typically consist of 3D convolutional layers, max-pooling layers, and fully connected layers for classification tasks. Additionally, 3D CNNs can represent the 3D volume of each time point as a 1D vector for further analysis (Dong *et al*., 2020b). In Dong *et al*. (2020b), a deeper architecture with several residual blocks—each containing two 3D convolutional layers and up/down pooling layers—was employed, achieving a depth of 34 convolutional layers, showing the improved performance compared with shallow models.

#### 4D convolutional neural networks

**Figure 3:**
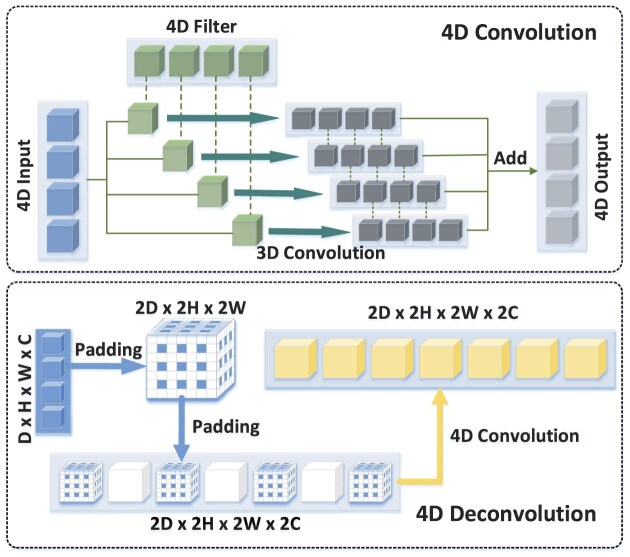
The illustration of 4D convolution/deconvolution operation when applied to fMRI data (Jiang *et al*., 2023).

4D CNNs integrate both spatial and temporal information, making them powerful for analyzing dynamic volumetric data such as fMRI data. Figure 3 illustrates the 4D convolution and deconvolution operations, which extend traditional 3D convolutions by incorporating a temporal dimension. The 4D convolution processes 4D fMRI data across spatial and temporal axes, enabling the extraction of spatiotemporal features, while the 4D deconvolution reconstructs feature maps to restore original spatial–temporal resolutions. 4D CNNs have been used to characterize FBNs (Jiang *et al*., 2023) and identify abnormal patterns in ASD (Liu *et al*., 2023a). Additionally, 4D CNNs have been employed to model both the spatial and temporal patterns of targeted FBNs (Yan *et al*., 2021b) and to classify attention deficit/hyperactivity disorder (ADHD) based on fMRI data (Mao *et al*., 2019). Besides directly applying 4D convolutions, some studies process 4D fMRI data by using 3D CNNs for spatial information and 1D CNNs for temporal information. For example, a spatio-temporal CNN (ST-CNN) was proposed to identify the default mode network (DMN) from fMRI data (Zhao *et al*., 2018c).

### Recurrent neural networks

**Figure 4:**
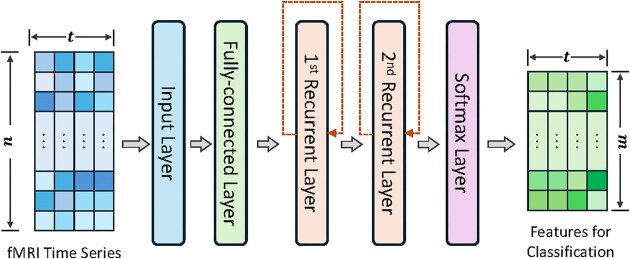
The architecture of the DSRNN model proposed in Wang *et al*. (2018). It consists of a fully connected layer to extract activated brain regions, followed by two recurrent layers to capture temporal dynamics. A softmax classifier is finally applied for brain state recognition.

RNNs are highly effective at modeling sequential data due to their capability to retain information from previous inputs via internal states. This makes them particularly well-suited for modeling the temporal dependencies in fMRI data. In the field of brain functional mapping, RNNs have been extensively applied to identify evolving patterns of brain activity over time (Wang *et al*., 2018) and to decompose FBNs (Cui *et al*., 2019; Li *et al*., 2019, 2021a, b; Qiang *et al*., 2021). Typically, each 3D fMRI volume at a given time point is embedded as a vector using a fully connected layer, and recurrent models, such as LSTM (Hochreiter and Schmidhuber, 1997), are employed to model the temporal dependencies of these vectors (Fig. 4). LSTM networks are particularly effective in handling long-range dependencies and mitigating the vanishing gradient problem commonly encountered in traditional RNNs.

### Transformer and self-attention

Transformer was designed to handle sequential data by utilizing self-attention mechanisms, which allow them to weigh the importance of different parts of the input sequence independently. Unlike traditional recurrent models, transformers can process entire sequences in parallel, making them highly efficient and effective for tasks involving long-range dependencies. In the context of fMRI data modeling, transformers have emerged as powerful tools for capturing complex patterns of brain activity (Dong *et al*., 2020c; Zhao *et al*., 2022; Mao *et al*., 2024). In Dong *et al*. (2020c), a self-attention mechanism was utilized to model the temporal relationship of each time point's representation. In Zhao *et al*. (2023a), the multi-head self-attention (MSA) module in the Transformer model demonstrated better representation ability than LSTM in modeling fMRI data. Transformer has also been employed to encode the brain function in a latent space as dense embedding vectors (Zhao *et al*., 2022, 2023c). In Kim *et al*. (2023), a SwiFT (Swin 4D fMRI Transformer) model was proposed to learn brain dynamics directly from 4D fMRI volumes to predict sex, age, and cognitive intelligence.

### Deep belief networks

**Figure 5:**
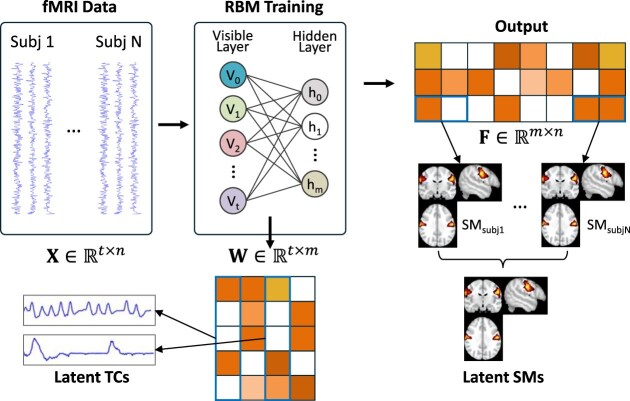
The RBM model proposed in Hu *et al*. (2018) interprets the weights as latent time courses, with its output representing spatial maps.

DBNs are built using layers of restricted Boltzmann machines (RBMs) (Hinton and Salakhutdinov, 2006), which are shallow, two-layer neural nets. Each RBM in a DBN learns to model the probability distribution of its inputs, and the layers are stacked such that the outputs of one RBM serve as the inputs to the next (Hinton, 2009). This hierarchical structure allows DBNs to capture complex, high-level features of the input data by learning progressively abstract representations. In brain function representation, both RBMs and DBNs have been widely used (Huang *et al*., 2016; Li *et al*., 2018b; Hu *et al*., 2018, 2018a; Dong *et al*., 2019; Zhang *et al*., 2019a, b, c; Qiang *et al*., 2020b; Ren *et al*., 2021; Pang *et al*., 2022a). For instance, RBMs were initially proposed to learn latent sources (components) of input fMRI data, where each latent component is associated with a time course (Huang *et al*., 2016). The number of units in the visible layer corresponds to the number of fMRI time points, while the hidden layer units represent latent sources. Using these learned latent components, spatial maps can be derived by multiplying the feature matrix with the input fMRI signals (Fig. 2b). Similarly, in Hu *et al*. (2018), the visible units represent the fMRI time points, the hidden units capture the latent components, and the weights between the layers correspond to the time courses (Fig. 5). The model's output is then interpreted as spatial maps.

In Li *et al*. (2018a), a two-layer DBN was applied to fMRI blind source separation, where the overall latent factors were derived by linearly combining the weight matrix from all hidden layers. Zhang *et al*. (2019b) adopted a three-layer DBN and using the NAS to optimize the number of hidden units in the DBN. Later, a volumetric sparse DBN was proposed (Dong *et al*., 2019), which differed from earlier approaches (Hu *et al*., 2018; Li *et al*., 2018a) by using visible units corresponding to the number of voxels in the fMRI scans. Each row of the weight matrix was mapped back into the original 3D brain image space and interpreted as an FBN. More recently, a novel prior knowledge-guided DBN (PKG-DBN) was introduced to address the limitations in hierarchical FBN analysis (Pang *et al*., 2022b). This approach enforces part of the time courses learned from the DBN to be task-related (either positively or negatively), with the remaining components being linear combinations of the task-related elements.

### Graph neural networks

GNNs are designed to operate on graph-structured data (Scarselli *et al*., 2008; Wu *et al*., 2020). Unlike traditional neural networks that work on Euclidean data such as images or sequences, GNNs leverage the structure of graphs to propagate information between nodes, enabling the learning of both node-level and graph-level representations. In fMRI data representation, different brain regions can be represented as nodes and their functional connections as edges, forming a graph that captures the brain's connectivity structure. For example, in Yuan *et al*. (2018), different group-wise FBNs are modeled as nodes and their interactions are modeled as edges, and a time-evolving graph model was then utilized to represent dynamic change of those interactions over time. In Zhang *et al*. (2021a), a multi-layer graph convolution network (GCN) was adopted to simultaneously model brain structure and function in mild cognitive impairment (MCI). Gadgil *et al*. (2020) proposed a spatial-temporal GCN (ST-GCN) to model the non-stationary nature of functional connectivity where each region of interest is represented as the node in the spatiotemporal graph.

### Neural architecture search

NAS is an automated method for designing neural network architectures that aims to optimize their performance for specific tasks (Zoph and Le, 2016; Liu *et al*., 2018). Unlike traditional approaches where architectures are manually designed by experts, NAS uses algorithms to explore a vast space of potential architectures and automatically identify the most effective designs.

**Figure 6:**
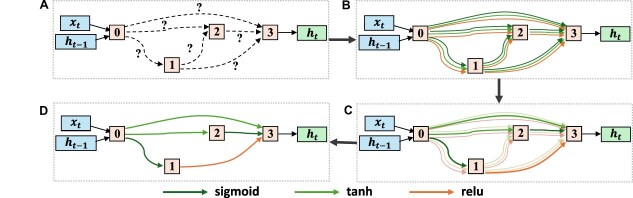
The differentiable NAS framework, as illustrated in Li *et al*. (2021a), automatically searches for the connections between computational nodes within an RNN cell and optimizes the activation function.

For brain function representation, one of the key advantages of NAS is its ability to systematically explore a wide range of architectures, including unconventional designs that may be more aligned with the complex nature of brain function. NAS has been applied to search the structure of RNN cells for decomposing the spatiotemporal FBNs from fMRI (Li *et al*., 2020, 2021a, b; Dai *et al*., 2022), the optimal CNN structure for fMRI signal classfication (Dai *et al*., 2020), and the number of units in each layer of DBNs (Zhang *et al*., 2019b; Qiang *et al*., 2020a; Ren *et al*., 2021; Pang *et al*., 2022a) for identifing FBNs. Compared to manually crafted network architectures, NAS-optimized neural networks have demonstrated improved performance in these tasks. Among these works, different searching strategies are employed to search the optimal network structures such as evolutionary algorithms (Li *et al*., 2020, 2021b; Zhang *et al*., 2019b; Qiang *et al*., 2020a; Ren *et al*., 2021), a gradient-based method (Li *et al*., 2021a)(Fig. 6), a graph-based method (Dai *et al*., 2022), and adaptive boosting technique (Dai *et al*., 2020).

## Learning paradigms for fMRI-based brain function mapping

For fMRI-based brain function mapping, various learning paradigms have been employed. Supervised learning involves training models on labeled datasets, enabling the accurate identification and classification of brain states or FBNs. However, due to the limited availability of annotations, unsupervised learning has been widely adopted to uncover hidden structures and patterns embodied in fMRI data. More recently, self-supervised learning has emerged as a promising approach, allowing models to learn from large-scale unlabeled data through surrogate tasks, thereby reducing the reliance on extensive labeling while producing high-quality representations of neural activity.

### Supervised learning approaches

Supervised learning approaches rely on the availability of labeled data, where the goal is to map input, such as fMRI data, to known output labels, such as brain states or FBNs derived from other methods. The input and output labels for supervised learning tasks vary depending on the specific objective (Ren *et al*., 2017; Zhao *et al*., 2018c, 2019; Wang *et al*., 2018; Yu *et al*., 2022, 2023c). For example, while SDL methods can reconstruct hundreds of FBNs, accurately classifying these networks remains challenging due to their intrinsic variability, noise, and lack of direct correspondence across subjects. Deep learning-based approaches can help address these challenges. Ren *et al*. (2017 and Zhao *et al*. (2017b) used a 3D CNN was to process the volumetric representations of FBNs and predict their respective classes. To mitigate the need for large labeled datasets, Zhao *et al*. (2018a) proposed an iteratively optimized CNN (IO-CNN) framework with automatic weak label initialization, reducing reliance on extensive annotated data.

For modeling fMRI data, Zhao *et al*. (2018c) introduced a deep ST-CNN to model 4D fMRI data, using the data as input and the spatial patterns and temporal dynamics of the default mode network (DMN) as supervisory labels. Similarly, in Yan *et al*. (2021a, 2022), a multi-head guided attention GNN (multi-head GAGNN) was developed to simultaneously capture the spatiotemporal patterns of multiple brain functional networks. The input in these models is the 4D fMRI data, with the supervision labels being the 10 resting state networks (RSNs) and their associated temporal patterns. In Cui *et al*. (2018a, b), a deep RNN (DRNN) framework was proposed to model FBNs from tfMRI data. In this approach, the task design stimulus curves for each subject are gathered into a stimulus matrix as the input, while the whole-brain tfMRI signals are aggregated into a large signal matrix as the output.

For brain state recognition, Wang *et al*. (2018) proposed a five-layer deep sparse RNN (DSRNN) to accurately classify brain states. In this model, the input consists of fMRI data at specific time points, and the output is the corresponding brain state, making it a classification task. In Liu *et al*. (2019) and Wang *et al*. (2023), fMRI signal representation is formulated as a classification task, using a 1D CNN model to distinguish between gyral and sulcal fMRI time series. By analyzing the weights in the fully connected layer responsible for predictions, they identified filters corresponding to gyri and sulci, which were then studied to uncover the distinct features of these signals. Building on this, Dai *et al*. (2020) demonstrated that CNN models initially developed for two-class (gyral vs sulcal) classification could be further optimized to handle more complex tasks, such as three-class classification (three-hinge gyral vs two-hinge gyral vs sulcal).

### Unsupervised learning approaches

Unsupervised learning involves training models on data without labels and annotations, allowing them to discover hidden structures and patterns independently. This approach is especially valuable for brain function mapping where the “ground truth” for individual/group brain function is lacking.

### Autoencoder

Autoencoders are a type of artificial neural network designed to learn efficient, compressed representations of input data. They consist of two main parts: an encoder, which compresses the input data into a latent space, and a decoder, which reconstructs the input from the compressed representation. Autoencoders are particularly useful for brain function mapping because they can reduce the dimensionality of the data while preserving important features, making it easier to analyze and interpret complex neural patterns in fMRI data. Spatially, fMRI scans consist of a vast number of voxels, each representing a small volume of brain tissue. Analyzing these voxels individually is impractical due to their sheer number and the intricate relationships between different brain regions. These regions often exhibit similar functional characteristics, which suggest underlying patterns and groupings that are not explicitly labeled in the data. Autoencoders can address this challenge by compressing the spatial dimensions of the data, enabling the identification of these meaningful patterns. Through this compression, autoencoders uncover latent structures within the brain, such as FBNs, without requiring labeled data, making them invaluable for exploratory analysis.

**Figure 7:**
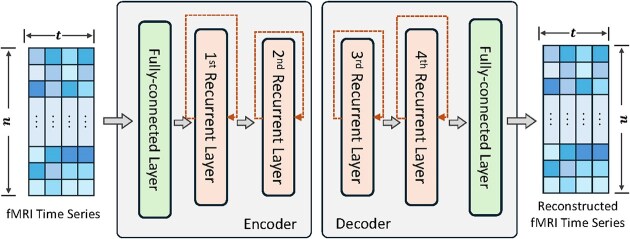
The deep sparsity recurrent autoencoder (DSRAE) proposed in Li *et al*. (2019) compresses fMRI volumes by reducing the number of voxels in successive layers. Initially, the fully connected layer reduces the voxel count to 128, followed by further reduction to 64 in the first recurrent layer, and finally to 32 in the second recurrent layer. This spatial compression enables the model to uncover latent patterns, such as functional brain networks (FBNs), from the vast number of fMRI voxels.

Various types of autoencoders have been employed for brain function mapping, each tailored to different aspects of the data. Convolutional autoencoders (CAEs) are particularly effective for handling both spatial and temporal data. 1D convolutional layers were adopted in CAE models to capture the characteristics of fMRI time series (Huang *et al*., 2017, 2018; Wang *et al*., 2019; Makkie *et al*., 2019; Zhao *et al*., 2021, 2024). A 3D CAE model was used to extract spatial brain network features (Zhao *et al*., 2017a) and learn representations for 3D fMRI volumes (Dong *et al*., 2020b). Recurrent autoencoders (RAEs), on the other hand, are well-suited for modeling temporal dynamics in fMRI data (Li *et al*., 2019; Cui *et al*., 2019; Li *et al*., 2020, 2021b; Qiang *et al*., 2021; Dai *et al*., 2022). These models typically use fully connected layers to reduce spatial dimensions, followed by recurrent layers that capture temporal dependencies across time steps (Fig. 7). Variational autoencoders (VAEs) introduce a probabilistic approach to the latent space, enabling the generation of new data samples that reflect the variability in brain function. This is useful for both data augmentation and understanding the distribution of neural activity (Qiang *et al*., 2020a, 2021). Additionally, attention-based (Liu *et al*., 2023c, 2024a, b) and transformer-based methods (Dong *et al*., 2020c; Zhao *et al*., 2022, 2023c) have been incorporated into autoencoders to further enhance the representation of brain function data.

### Generative adversarial networks

Generative adversarial networks (GANs) consist of two neural networks: a generator and a discriminator, which are trained simultaneously through an adversarial process. The generator network creates synthetic data that mimic the distribution of the training data, while the discriminator network attempts to distinguish between real and generated data. This adversarial training setup drives both networks to improve continuously, leading to a generator capable of producing data that closely resemble the original.

In brain function mapping, GANs can capture the underlying distribution of fMRI data and generate new samples that reflect the variability and complexity of brain function. In Dong *et al*. (2020a), the trained discriminator encodes representations of real fMRI data, with the features extracted by the discriminator interpreted as functional brain networks. A recurrent Wasserstein GAN (RWGAN) was proposed in Qiang *et al*. (2022) to learn brain representations from volumetric fMRI data. In this model, the discriminator acts as a deep feature extractor, while the generator produces high-quality synthetic data for fMRI augmentation. Additionally, the VAE–GAN framework, which combines a VAE with a GAN, was introduced for functional brain network identification and fMRI data augmentation (Qiang *et al*., 2023).

### Self-supervised learning approaches

Self-supervised learning has recently gained attention in fMRI-based brain function analysis, offering a promising alternative that allows models to learn meaningful representations from unlabeled data with surrogate tasks. For fMRI data representation, these tasks often include predicting missing data points (Ortega Caro *et al*., 2024), reconstructing temporal sequences (Yang *et al*., 2024), or generating future brain states based on past activity. By leveraging large amounts of unlabeled fMRI data, self-supervised learning has shown great potential in learning robust, generalizable representations that can later be fine-tuned for specific tasks, such as brain state recognition or functional network identification.

Building on this promising paradigm, recent works have made significant advancements in applying self-supervised learning to brain function mapping through a variety of innovative approaches. In Thomas *et al*. (2022), sequences of brain activity are represented similarly to text in natural language processing (NLP), and self-supervised learning frameworks are introduced by applying techniques such as sequence-to-sequence autoencoding, causal language modeling, and masked language modeling to uncover spatiotemporal patterns in brain dynamics from large-scale neuroimaging data. Inspired by masked image modeling (MIM) (He *et al*., 2022), BrainLM introduces a Transformer masked autoencoder pre-trained to reconstruct masked signals in fMRI recordings by leveraging both visible and masked tokens (Ortega Caro *et al*., 2024). The pre-trained model can then be fine-tuned for specific tasks or applied directly for zero-shot inference. Similarly, BrainMAE takes a region-aware approach by randomly masking regions of interest (ROIs) within fMRI segments and challenging the model to reconstruct these masked signals, thus learning functional relationships between brain regions (Yang *et al*., 2024). In Malkiel *et al*. (2022), a Transformer framework for fMRI (TFF) was proposed, utilizing self-supervised pre-training to reconstruct 3D fMRI volumes. After pre-training, the model is fine-tuned for specific tasks such as age prediction and schizophrenia diagnosis. SwiFT (Swin 4D fMRI Transformer) utilizes contrastive learning for pre-training (Kim *et al*., 2023), employing two types of loss functions: instance contrastive loss, which distinguishes between fMRI sub-sequences from different subjects, and local–local temporal contrastive loss, which differentiates fMRI sub-sequences from different timestamps within the same subject.

## Trends, challenges, and opportunities

As advancements continue to unfold, new trends, challenges, and opportunities are emerging with the potential to shape the future of brain function mapping and neuroimaging research. These developments have the potential to drive significant improvements in how brain activity is represented, modeled, and understood. In this section, we highlight three critical areas of growth: the development of fMRI embeddings, which aim to create more compact and informative representations of brain activity; foundation models, which offer a generalized framework for applying learned representations across multiple tasks; and brain-inspired AI, which seeks to bridge the gap between human cognition and artificial intelligence, enhancing the capabilities of AI systems.

### fMRI embedding

**Figure 8:**
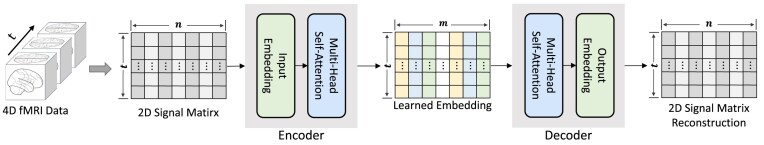
The fMRI embedding framework proposed by Zhao *et al*. (2023c) restructures 4D fMRI signals into 2D matrices. These matrices are then processed through linear transformations and transformer layers, effectively reducing spatial dimensionality while capturing temporal relationships within the data.

Traditional brain functional mapping and representation, such as FBNs, often rely on methods like ICA and SDL to extract spatial and temporal patterns from fMRI data. The whole brain's functional profile can be viewed as discrete, one-hot embeddings which only encode the presence or absence of specific FBNs. Such representations fail to capture the variability, complexity, and inter-subject differences inherent in brain activity patterns across diverse populations and dynamic time points. Recently, researchers have started applying embedding techniques to profile the whole brain's function representation as continuous vectors (Zhao *et al*., 2022; Thomas *et al*., 2022; Zhao *et al*., 2023a, c). Previous studies (e.g. Casanova *et al*., 2021) explored the concept of embedding brain function by linearly projecting FBNs into lower-dimensional spaces, reducing the dimensionality of FBNs to a more compact representation. However, it remains limited by the challenges previously discussed. Rather than treating brain activity as isolated, independent components like FBNs, several studies on fMRI embeddings represent functional brain patterns as dense vectors in a continuous space. In Zhao *et al*. (2022, 2023c), an unsupervised embedding framework was proposed using an encoding–decoding architecture, where 4D fMRI signals are rearranged into 2D matrices and passed through linear transformation and transformer layers to reduce spatial dimensionality while exploring temporal relationships (Fig. 8). By learning from large-scale fMRI datasets, these models can capture both the regularity and variability of brain activity across individuals and time points, resulting in a more compact and meaningful representation. This approach enables better comparisons between brain states, more accurate brain function mapping, and improved performance on downstream tasks such as brain state prediction and network identification.

Building on these advancements, future work in fMRI embeddings can explore several promising directions. First, improving the interpretability of these embeddings will be essential for translating insights into practical applications in neuroscience and clinical settings. A particularly promising approach is the incorporation of biologically informed constraints or priors into embedding models, which could generate representations that more closely align with known brain structures and functions. Additionally, future studies could investigate the integration of multimodal data—such as electroencephalography (EEG) or magnetoencephalography (MEG)—into the embedding framework, creating richer, more comprehensive models of brain activity that capture both spatial and temporal dynamics across different imaging modalities. Lastly, self-supervised learning techniques hold significant potential in this area. These approaches can help models learn more robust and generalizable representations from unlabeled and noisy data, further enhancing their effectiveness for brain function mapping and other neuroimaging tasks.

### Brain foundation models

Foundation models have emerged as a transformative approach in fields like NLP and computer vision (Radford *et al*., 2018; Dosovitskiy *et al*., 2020; Oquab *et al*., 2023; Dubey *et al*., 2024). These models are pre-trained on large-scale datasets and can be fine-tuned or directly applied for a variety of downstream tasks. The key benefit of foundation models lies in their ability to learn rich, generalizable features from large amounts of data, making them highly adaptable to new tasks with strong performance.

Inspired by the success of foundation models in language and vision, researchers have begun exploring the brain foundation model for diverse brain-related tasks with large-scale pre-training (Thomas *et al*., 2022; Ortega Caro *et al*., 2024; Yang *et al*., 2024; Kim *et al*., 2023). For example, Thomas *et al*. (2022) proposed a framework that models the dynamics of brain activity by treating sequences of brain signals similarly to how sequences of text are modeled in NLP. The study demonstrated that pre-trained models significantly outperformed others in downstream adaptation tasks on two benchmark mental state decoding datasets. Brain Language Model (BrainLM) is a foundation model for brain activity dynamics, trained on 6700 h of fMRI recordings using self-supervised masked-prediction training (Ortega Caro *et al*., 2024). It excels in both fine-tuning and zero-shot inference tasks, enabling the accurate prediction of clinical variables, identification of intrinsic functional networks, and generation of interpretable latent representations. BrainMAE is designed to learn representations directly from fMRI time-series data using a self-supervised masked autoencoding framework (Yang *et al*., 2024). It incorporates a region-aware graph attention mechanism to capture relationships between ROIs and effectively models the rich temporal dynamics of fMRI data. BrainMAE consistently outperforms baseline methods across various downstream tasks, including steady-state variable prediction and transient mental state decoding.

Despite these advancements, brain foundation models still face several challenges. One major issue is the limited availability of large-scale, high-quality fMRI datasets, which restricts the model's ability to generalize across diverse populations. To mitigate this limitation, researchers increasingly employ strategies such as transfer learning, data augmentation, and synthetic data generation. Transfer learning utilizes pre-trained neural networks, capturing generalizable features that can be efficiently fine-tuned to smaller, task-specific fMRI datasets, thus enhancing model performance (Zhang *et al*., 2018a; Svanera *et al*., 2019; Al-Hiyali *et al*., 2021). Data augmentation techniques, such as Gaussian noise and Mixup, expand dataset variability, strengthening the robustness and generalizability of models (Pei *et al*., 2022; Smucny *et al*., 2022). Additionally, synthetic data generation methods, such as GANs (Zhuang *et al*., 2019), VAEs (Qiang *et al*., 2021), and diffusion models (Hu et, al.), have demonstrated promise in creating realistic artificial fMRI samples that closely resemble brain activity, which can effectively alleviate the data limitation.

Another key area for improvement is interpretability—while these models offer powerful capabilities, gaining a deeper understanding of their decision-making processes is crucial for their application in both clinical and neuroscientific contexts. Additionally, further exploration of their potential clinical value is essential, as these models could offer new insights into diagnosis, treatment planning, and understanding of brain disorders in real-world medical scenarios. Addressing these challenges will be crucial to unlocking the full potential of brain foundation models in advancing neuroscience and healthcare applications.

### Brain-inspired AI

While current AI models have achieved remarkable success, they still lack the flexibility, adaptability, and efficiency demonstrated by biological neural networks (BNNs). Brain-inspired AI seeks to bridge this gap by drawing the inspirations from the human brain, such as the organizational and functional principles, to develop more efficient and powerful AI systems (Zhao *et al*., 2023d). Recent studies have shown that the responses of artificial neural networks (ANNs) to external stimuli closely mirror those of their biological counterparts in various domains, including visual (Zhao *et al*., 2023a; Yamins and DiCarlo, 2016; Kriegeskorte, 2015), auditory (Zhou *et al*., 2023; Li *et al*., 2023; Millet *et al*., 2022), and linguistic processing (Liu *et al*., 2023b; Caucheteux and King, 2022; Schrimpf *et al*., 2021). Additionally, the topology of ANNs are similar to BNNs (Du *et al*., 2023). This intriguing correspondence suggests that ANNs may evolve to process and organize information in ways similar to the human brain. Another group of works (Yu *et al*., 2023a; Zhao *et al*., 2023b; Lyu *et al*., 2024; Yu *et al*., 2024) have sought to incorporate brain organizational principles, such as core-periphery organization, directly into ANN architectures, leading to improved performance. Building on these advancements, researchers can leverage brain-inspired AI models to develop more precise and efficient brain mapping tools that capture the dynamic complexity of neural processes. In turn, these enhanced maps could provide critical insights to refine AI architectures and learning mechanisms, making them more flexible, adaptive, and context-aware—traits akin to the human brain. This synergy between neuroscience and AI holds great promise for breakthroughs in personalized healthcare, advanced cognitive computing, and brain–computer interfaces, paving the way for more intelligent and human-aligned AI technologies.

## Real-world applications and prospective impact

In this section, we focus on the real-world applications and prospective impact of brain function mapping. Advances in mapping the intricate processes of the brain hold significant promise for a range of fields, including neuroscience, mental health, and neurotechnology. For example, more accurate brain maps can facilitate early detection and treatment of neurological disorders such as Alzheimer's, improving patient outcomes. Additionally, detailed brain function mapping can enhance brain–computer interfaces, providing new avenues for assistive technologies that restore movement or communication capabilities in individuals with disabilities. As these techniques continue to evolve, they will play a crucial role in advancing both scientific understanding and practical applications in healthcare and beyond.

### Neural disorders and clinical applications

One of the most promising applications of brain function mapping lies in its potential to advance the diagnosis and treatment of neural disorders (Zhang *et al*., 2021b; Yu *et al*., 2023c). For example, the extracted spatiotemporal patterns of brain activities can be used to detect ADHD (Mao *et al*., 2019; Dong *et al*., 2020c; Qiang *et al*., 2020a). The 3D CNNs proposed by Zhao *et al*. (2018b) have demonstrated the ability to differentiate ASD from healthy controls, offering a valuable tool for early diagnosis. Similarly, by integrating brain functional profiles, Zhang *et al*. (2021a) successfully differentiated MCI patients from elderly normal controls, a critical step in managing early-stage cognitive decline. Moreover, Zhang *et al*. (2023) leveraged brain functional connectivity to learn clinical group-related feature vectors, achieving higher accuracy in classifying Alzheimer's disease. These advancements highlight the potential of brain function mapping to transform the clinical landscape, enabling earlier diagnoses and more targeted interventions for neurological disorders.

### Neurosurgical practices and clinical decision-making

Brain function mapping holds great promise for neurosurgical applications, facilitating precise surgical interventions and improving patient outcomes. Accurate preoperative brain functional mapping can guide surgical procedures by identifying and preserving critical functional regions associated with language, vision, and motor activities, thereby minimizing postoperative neurological deficits. For example, detailed, patient-specific brain maps allow neurosurgeons to precisely visualize essential cortical and subcortical regions and inform surgical trajectories and help surgeons effectively avoid damaging eloquent areas during operations. Brain function mapping also enables predictive neurosurgical outcomes. For example, individualized brain functional mapping can be utilized to forecast individual patient responses to surgical treatments, estimate recovery trajectories, and predict the risk of postoperative complications, ultimately aiding neurosurgeons in selecting the most effective therapeutic strategies.

### Advancing neuroscientific understanding

Beyond clinical applications, brain function mapping plays a pivotal role in advancing neuroscientific understanding. The represented patterns of brain activity uncover new insights into brain function and organization. For instance, a group of have systematically examined the characteristics of gyri and sulci fMRI signals, suggesting distinct functional roles for gyri and sulci and providing insights into their underlying mechanisms (Zhang *et al*., 2018b; Liu *et al*., 2019; Zhao *et al*., 2021; Wang *et al*., 2023, 2024). In addition, a twin transformer framework proposed by Yu *et al*. (2023b) effectively disentangles the spatiotemporal patterns of gyri and sulci, revealing that these brain structures may collaborate in a core–periphery network manner. These findings deepen our understanding of the brain's structural and functional complexity and highlight how advanced models can elucidate the brain's intricate dynamics. This expanding body of work not only sheds light on previously unexplored brain mechanisms but also opens new avenues for future research in neuroscience.

### Decoding brain activity

Another exciting application of brain function mapping is the ability to decode specific mental states, sensory perceptions, and even language directly from brain activity. AI models have made significant strides in decoding visual perception from brain signals, allowing researchers to reconstruct images based on what individuals are seeing or imagining. For instance, using fMRI data, these models can predict visual stimuli with impressive accuracy (Fang *et al*., 2020; Chen *et al*., 2023; Takagi and Nishimoto, 2023). Similarly, decoding language from brain activity has gained traction (Gauthier and Ivanova, 2018; Affolter *et al*., 2020), with AI-driven methods showing promise in translating neural signals into speech or text (D´efossez *et al*., 2023; Berezutskaya *et al*., 2023), offering a pathway for enhancing communication for individuals with speech impairments.

These advances hold great potential for brain–computer interfaces (BCIs) by enabling direct neural control of assistive devices, thereby enhancing mobility, communication, and overall independence for individuals with disabilities. BCIs leveraging brain decoding could enable paralyzed individuals to control external devices or communicate by translating their thoughts into actions or speech. Moreover, sensory decoding, such as interpreting auditory or tactile information from brain signals, could open doors to new rehabilitation tools for people with sensory deficits, creating new opportunities for restoring lost functions. As these innovations continue to evolve, they hold the promise of not only improving the functionality and autonomy of people with disabilities but also advancing our understanding of the brain's capacity to adapt and interact with the external world in ways previously thought impossible.
